# Causal association between matrix metalloproteinases and diabetic neuropathy: a two-sample Mendelian randomization study

**DOI:** 10.3389/fendo.2024.1429121

**Published:** 2025-01-14

**Authors:** Chao Bai, Wenwen Yang, Guangwei Qi, Liuyu Yang, Qingrui Wu, Jieguang Peng, Ning Wang, Tao Liu

**Affiliations:** ^1^ Vascular and Thyroid Surgery, The First Affiliated Hospital of Xinjiang Medical University, Urumqi, Xinjiang, China; ^2^ Postdoctoral Research Station of Public Health and Preventive Medicine, Xinjiang Medical University, Urumqi, Xinjiang, China; ^3^ Department of Clinical Nutrition, The First Affiliated Hospital of Xinjiang Medical University, Urumqi, Xinjiang, China; ^4^ The First Department of General Internal Medicine, State Key Laboratory of Pathogenesis, Prevention and Treatment of High Incidence Diseases in Central Asia, the First Affiliated Hospital of Xinjiang Medical University, Urumqi, China; ^5^ School of Public Health, Xinjiang Medical University, Urumqi, Xinjiang, China

**Keywords:** diabetic neuropathy, Mendelian randomization, MMP-2, MMP-16, GWAS

## Abstract

**Objective:**

Diabetic neuropathy (DN), a common and debilitating complication of diabetes, significantly impairs the quality of life of affected individuals. While multiple studies have indicated changes in the expression of specific matrix metalloproteinases (MMPs) in patients with DN, and basic research has reported the impact of MMPs on DN, there is a lack of systematic research and the causal relationship remains unclear. The objective of this research is to investigate the casual relationship between MMPs and DN through two-sample Mendelian randomization (MR).

**Methods:**

Data for this investigation were derived from genome-wide association studies (GWAS) of MMPs and DN. For the analysis using two-sample MR, methods such as inverse variance weighted (IVW), weighted median, weighted mode, and MR-Egger were utilized, with IVW serving as the primary measure for determining causative impacts. To evaluate the analysis’ heterogeneity and potential pleiotropy, sensitivity examinations including MR-PRESSO analysis, Cochran’s Q test, and the leave-one-out test were conducted.

**Results:**

IVW analysis revealed that genetically decreased serum MMP-2 level were causally associated with a high risk of DN (OR = 0.88, 95% CI: 0.79-0.99, P = 0.026). Genetically elevated serum MMP-16 level were causally associated with a high risk of DN (OR = 1.15, 95% CI: 1.01-1.32, P = 0.038). Genetic prediction results showed no causal association between other MMPs (MMP14/17/9/12/7/3) and DN. Sensitivity analyses showed no significant heterogeneity or pleiotropy.

**Conclusion:**

In summary, this research uncovered a genetic causal relationship between heightened MMP-16 levels and reduced MMP-2 concentrations, and DN risk. These discoveries offer new perspectives on the role of MMPs in DN etiology and establish a foundational premise for further investigations into MMP-targeted therapeutic interventions.

## 1 Introduction

With the growing prevalence of obesity and an aging population, diabetes has seen a steady increase over the past two decades. By 2019, the global age-standardized prevalence rate reached approximately 5,282.9 per 100,000 people, with a corresponding mortality rate of about 18.5 per 100,000 (1). Diabetic neuropathy (DN) stands as the most widespread and clinically demanding complication associated with diabetes, affecting half of those diagnosed. This condition notably heightens disability rates, profoundly impacts the quality of life and treatment options for patients, substantially increases the disease burden, and escalates healthcare costs. While DN might be linked to factors such as insulin signaling, genetic predispositions, environmental influences, and obesity, specific treatments targeting its pathogenesis are still lacking. Matrix metalloproteinases (MMPs) are a group of enzymes capable of degrading extracellular matrix proteins, playing key roles in extracellular matrix remodeling, inflammatory responses, and cell signaling (2). These enzymes play pivotal roles in numerous physiological processes and pathological conditions, including diabetes and its associated neuropathic complications. Recent research highlights the significant influence of MMPs on the development and progression of DN (3). MMPs are particularly noteworthy for their ability to degrade the extracellular matrix of the peripheral nervous system, positioning them as potential molecular links between diabetes and neuropathy.

Clinical findings underscore the importance of MMPs in diabetes, revealing that MMP-14 is notably overexpressed in diabetic patients and is intricately linked to the onset of diabetic complications (4). In the context of DN, MMP-9 has been identified as a promising biomarker for type 1 DN (5), while elevated MMP-1 levels in type 2 diabetes have been linked to increased collagenase activity in Schwann cells—a key factor contributing to the development of DN (6). Despite the growing body of evidence connecting MMPs with DN, the conclusions of these studies remain tentative. The primary limitations stem from generally small sample sizes, the presence of confounding factors, and the potential for reverse causality in observational studies. This underscores the need for more robust, large-scale research to establish a definitive causal relationship between MMPs and DN, further exploring their role as therapeutic targets in mitigating diabetic complications.

Mendelian Randomization (MR) is a method that uses genetic variants as IVs to assess the causal relationship between exposures and diseases (7). The advantage of this method is that it can reduce the problems of confounding factors and reverse causality that cannot be overcome in traditional observational studies, providing more reliable evidence for causal inference. Two-sample MR analysis, using exposure and outcome data from different studies, further increases the statistical power of the analysis and reduces the impact of measurement errors. Therefore, this study aims to explore the causal relationship between MMP levels and the risk of DN using the two-sample MR method, aiming to provide more effective prevention and treatment methods for DN patients, thereby improving patients’ prognosis and quality of life.

## 2 Methods

### 2.1 Study design

**Figure 1:**
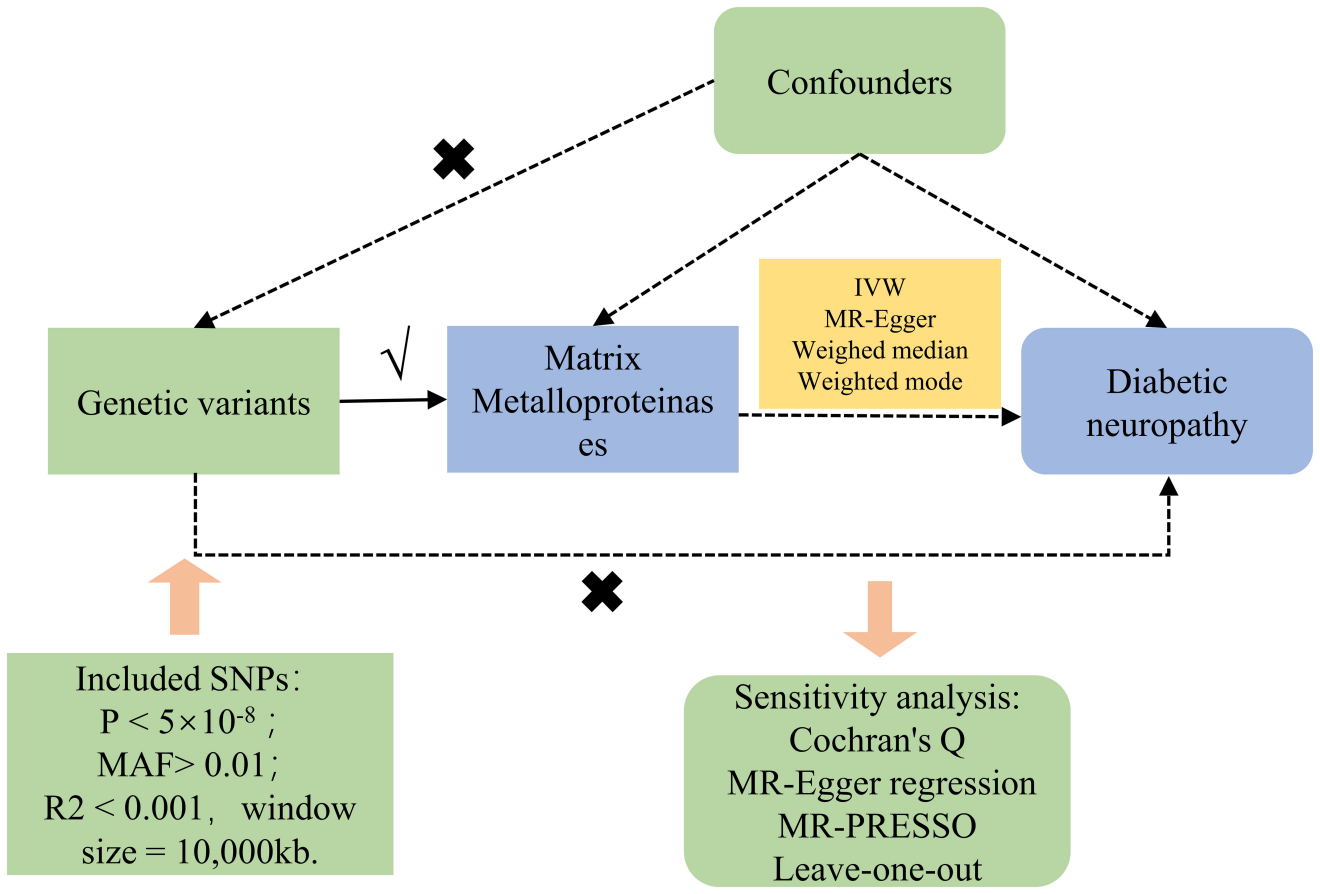
Study design and workflow.

**Figure 1** concisely delineates the MR framework linking MMPs with DN. Single nucleotide polymorphisms (SNPs) derived from GWAS summary statistics served as instrumental variables (IVs) in our two-sample MR evaluation. The MR assessment adheres to three cardinal principles (8): ① Genetic variants must exhibit a strong correlation with the exposure; ② Genetic variants should not correlate with any confounders affecting the exposure-outcome relationship; ③ The impact of genetic variants on the outcome should be mediated solely through the exposure, excluding alternate biological routes. The acquisition of GWAS summary statistics from publicly accessible repositories eliminated the necessity for additional ethical approval.

### 2.2 Data source

GWAS summary data related to DN were obtained from the FINNGEN database (https://www.finngen.fi/en/access_results), which encompassing 2,444 cases and 249,480 controls; GWAS data concerning MMPs were sourced from the GWAS Catalog database (https://www.ebi.ac.uk/gwas/downloads/summary-statistics). The cohort sizes for MMP-14/16/17/9 were 3,301 (9), for MMP-12/7 were 1,301 (10), and for MMP-2/3 were 1,323 (9, 10). These data were specifically collected from European descent. Comprehensive details, including GWAS ID, is available in **Supplementary Table 1**.

### 2.3 Instrumental variable selection

Within this research, the chosen IVs need to adhere to specific criteria: ①Firstly, SNPs related to the genome-wide significance of MMPs were identified by screening for a threshold where the P-value was less than 5×10^-8^. In cases where there were insufficient SNPs meeting this stringent criterion for further analysis, the threshold was relaxed to a P-value of less than 5×10^-6^, allowing for the selection of additional SNPs (11). ② Select SNPs exhibiting a minor allele frequency (MAF) above 0.01; ③ Exclude any linkage disequilibrium (LD) among SNPs, applying a threshold of R^2^ below 0.001 and a window size set to 10,000 kb (12); ④ Substitute any IV lacking in the outcome data with another SNP that demonstrates a high degree of linkage disequilibrium (R^2^ exceeding 0.8) with the original IV; ⑤ Determine the F value for each SNP within the IV to evaluate the IV’s robustness, aiming to mitigate the influence of potential weak instrument variable bias. This determination utilizes the formula: F = R^2^*(N-2)/(1-R^2^), where R^2^ denotes the fraction of variance in exposure elucidated by the SNP in the IV, and an F value exceeding 10 is deemed satisfactory (13).

### 2.4 MR analysis

The primary analytic approach utilized in this study was the Inverse Variance Weighted (IVW) method, focusing on the exploration of the causal association between exposure and the risk of outcomes by deriving Odds Ratios (OR) and 95% Confidence Intervals (CI) (14). Moreover, to verify the integrity of the findings, methods such as MR-Egger (15), weighted median (16), and weighted mode (17) were also applied. The MR-Egger method, integrating an intercept term, facilitates the precise estimation of causative impacts, even when pleiotropic bias is present. Predicated on the premise that a majority of the IVs maintain validity, the weighted median approach examines the causal dynamics between exposure and outcome. The “TwoSampleMR” package was employed for all statistical evaluations (18). Outcome visualization was achieved via scatter diagrams and charts illustrating sensitivity analysis. Considering the investigation addressed four outcome variables, the False Discovery Rate (FDR) correction strategy was adopted for adjusting P-values associated with multiple tests, treating pFDR values below 0.05 as evidentially significant.

### 2.5 Sensitivity analysis

The aim of sensitivity analysis within MR studies is to unearth potential issues of pleiotropy. This investigation assessed the heterogeneity among chosen IVs via Cochran’s Q test, noting that a P-value exceeding 0.05 denotes minimal heterogeneity, which implies that variations among estimates of IVs are stochastic and exert negligible influence on the outcomes of IVW analysis (19). Additionally, due to the possibility that pleiotropy among genetic variations could distort the accuracy of effect association estimates, MR-Egger regression was employed to scrutinize for horizontal pleiotropy. An intercept term in MR-Egger regression that is near zero or not statistically significant signifies an absence of pleiotropy concerns (15). Moreover, this research incorporated the MR Pleiotropy Residual Sum and Outlier (MR-PRESSO) strategy, designed to detect and exclude potential outliers (SNPs with a P-value below 0.05), and subsequently reassess the causal connection, thereby amending for horizontal pleiotropy (20).

## 3 Results

### 3.1 Inclusion of IVs

In our research, we identified 88 IVs pertinent to MMPs (**Table 1**). The MR evaluation targeted MMP-12, MMP-14, MMP-16, MMP-17, MMP-2, MMP-3, MMP-7, and MMP-9 as the exposures, incorporating 3, 15, 13, 14, 14, 7, 6, and 16 SNPs correspondingly. Subsequent to matching the SNPs with the outcome of DN, unmatched SNPs in the summary data for the indicated MMPs were shown as follows: 1, 3, 1, 4, 7, 1, 2 and 1. All selected IVs demonstrated robust validity with a minimum F-statistic exceeding 10, which indicates a strong instrument and reduces the risk of weak instrument bias.

**Table 1:** The causal relationship between MMPs exposures and DN as outcomes using Mendelian randomization.

Outcome	Exposure	Significant of SNP	N.SNPs	Methods	OR (95% CI)	P
Diabetic neuropathy	Matrix metalloproteinase-17 levels	5*10^-6^	5	IVW	1.00 (0.88 - 1.15)	0.955
			5	MR-Egger	1.29 (0.93 - 1.79)	0.162
			5	Weighted Median	1.02 (0.85 - 1.23)	0.796
			5	Weighted Mode	1.03 (0.77 - 1.38)	0.859
			5	Multiplicative random effects	1.00 (0.90 - 1.12)	0.945
	Matrix metalloproteinase-9 levels	5*10^-6^	6	IVW	0.90 (0.81 - 1.00)	0.061
			6	MR-Egger	0.95 (0.77 - 1.17)	0.616
			6	Weighted Median	0.91 (0.78 - 1.06)	0.236
			6	Weighted Mode	0.92 (0.77 - 1.10)	0.362
			6	Multiplicative random effects	0.90 (0.82 - 0.99)	0.029
	Matrix metalloproteinase-3 levels	5*10^-6^	6	IVW	0.99 (0.87 - 1.13)	0.920
			6	MR-Egger	0.88 (0.60 - 1.28)	0.535
			6	Weighted Median	0.97 (0.87 - 1.09)	0.655
			6	Weighted Mode	0.96 (0.85 - 1.09)	0.593
			6	Multiplicative random effects	0.99 (0.87 - 1.13)	0.920
	Matrix metalloproteinase-2 levels	5*10^-6^	6	IVW	0.88 (0.79 - 0.99)	0.026
			6	MR-Egger	0.79 (0.57 - 1.10)	0.220
			6	Weighted Median	0.93 (0.82 - 1.05)	0.220
			6	Weighted Mode	0.94 (0.81 - 1.09)	0.427
			6	Multiplicative random effects	0.88 (0.79 - 0.99)	0.026
	Matrix metalloproteinase-12 levels	5*10^-6^	4	IVW	1.09 (0.98 - 1.21)	0.109
			4	Multiplicative random effects	1.09 (0.99 - 1.20)	0.079
	Matrix metalloproteinase-14 levels	5*10^-6^	3	IVW	0.91 (0.75 - 1.10)	0.317
			3	MR-Egger	0.82 (0.45 - 1.49)	0.530
			3	Weighted Median	1.01 (0.81 - 1.26)	0.918
			3	Weighted Mode	1.00 (0.76 - 1.31)	0.999
			3	Multiplicative random effects	0.91 (0.75 - 1.10)	0.317
	Matrix metalloproteinase-16 levels	5*10^-6^	6	IVW	1.15 (1.01 - 1.32)	0.038
			6	MR-Egger	1.15 (0.82 - 1.62)	0.439
			6	Weighted Median	1.19 (1.00 - 1.42)	0.047
			6	Weighted Mode	1.31 (0.95 - 1.81)	0.124
			6	Multiplicative random effects	1.15 (1.03 - 1.29)	0.014
	Matrix metalloproteinase-7 levels	5*10^-6^	7	IVW	1.02 (0.88 - 1.19)	0.764
			7	MR-Egger	0.88 (0.59 - 1.32)	0.606
			7	Weighted Median	1.00 (0.83 - 1.20)	0.962
			7	Weighted Mode	0.97 (0.76 - 1.23)	0.796
			7	Multiplicative random effects	1.02 (0.89 - 1.18)	0.749

MMP, matrix metalloproteinases; DN, diabetic neuropathy. (The same meaning as the following tables)

### 3.2 MR analysis results

**Figure 2:**
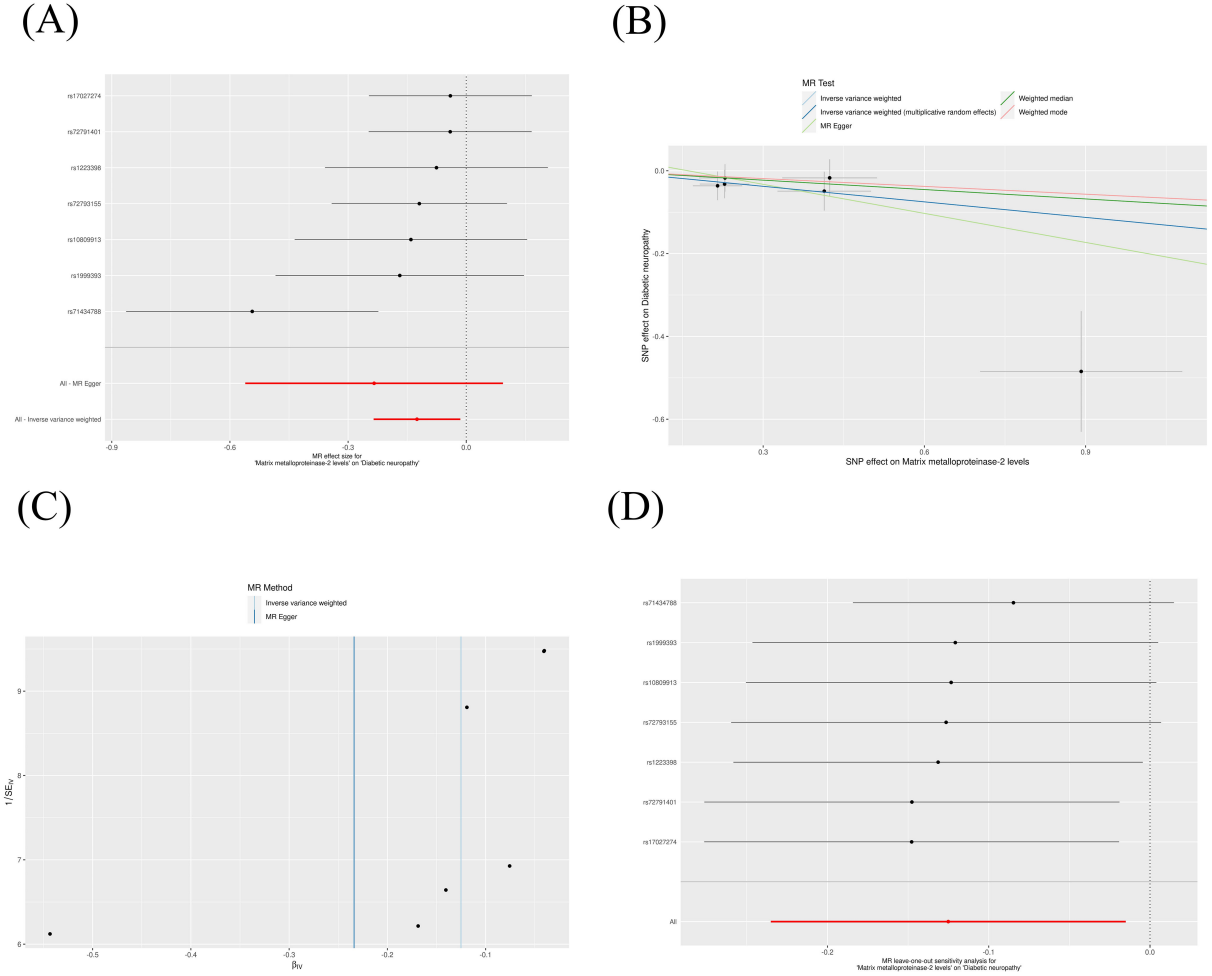
Scatter plot **(A)**, forest plot **(B)**, funnel plot **(C)** and leave-one-out analysis **(D)** of MMP-2 on DN. MMP, matrix metalloproteinases; DN, diabetic neuropathy. (The same meaning as the following figures).

**Figure 3:**
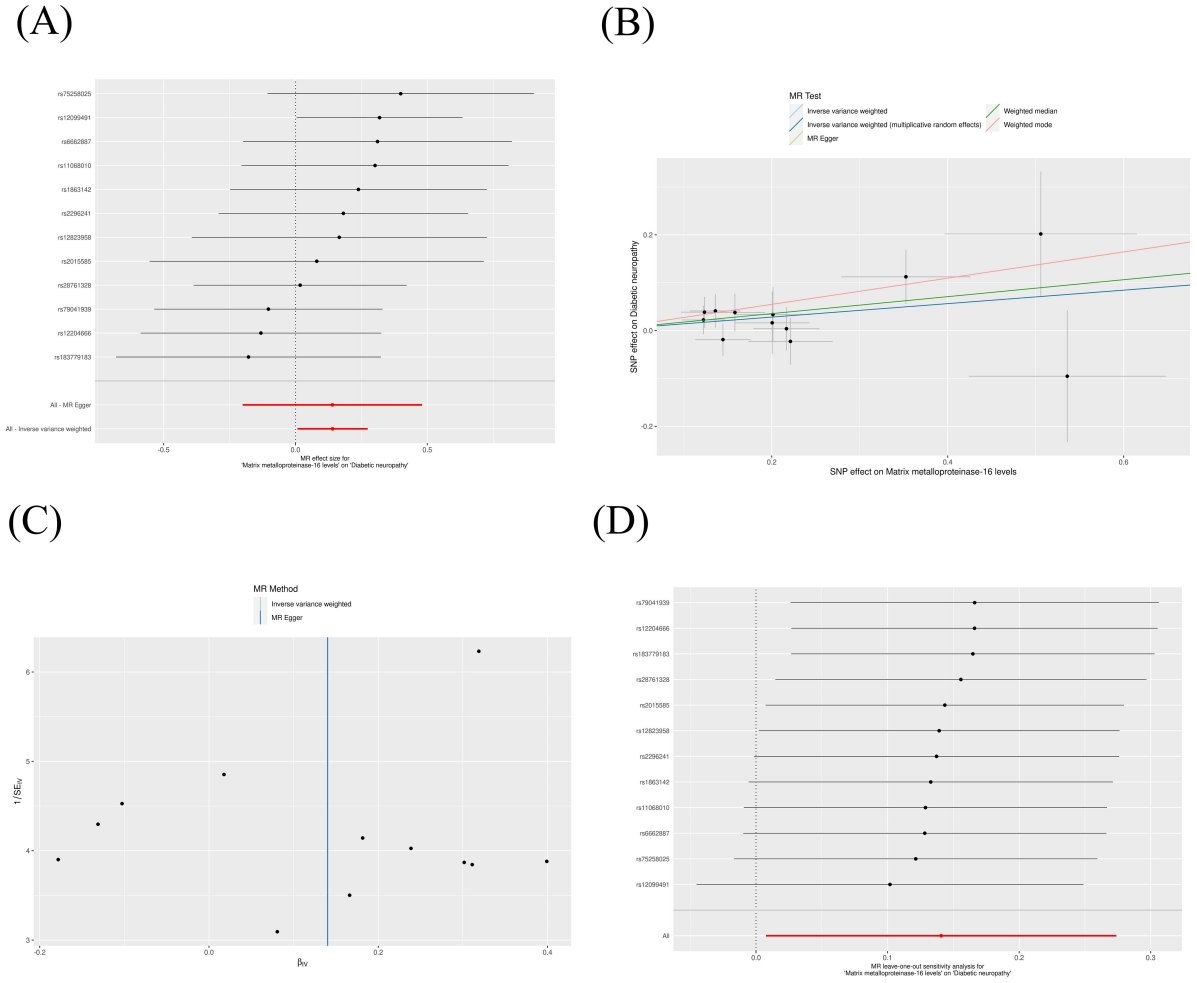
Scatter plot **(A)**, forest plot **(B)**, funnel plot **(C)** and leave-one-out analysis **(D)** of MMP-16 on DN.

The IVW method revealed that genetically decreased levels of serum MMP-2 were causally associated with an increased risk of DN (OR = 0.88, 95% CI: 0.79-0.99, P = 0.026). Conversely, elevated levels of serum MMP-16 were associated with an higher risk of DN (OR = 1.15, 95% CI: 1.01-1.32, P = 0.038), as detailed in **Table 1**. The outcomes from the weighted median, weighted mode, and MR-Egger methods are also elaborated in **Table 1**. To visualize the relationship between MMP-2/16 and DN, forest plots and scatter plots were utilized (**Figures 2A, B**, **3A, B**). Genetic predictions results showed no causal association between other MMPs and DN, with corresponding forest plots and scatter diagram outcomes presented in **Supplementary Figures 1**, **2**.

### 3.3 Sensitivity analysis

A suite of sensitivity assessments was conducted on the MR analysis outcomes linking MMPs with DN, including Cochran’s Q test, MR-Egger regression and MR-PRESSO test, and leave-one-out analysis, to confirm the robustness of the results and identifying any potential heterogeneity and pleiotropy. The Cochran’s Q statistic revealed no significant heterogeneity within the MR evaluation (P > 0.05). Additionally, the MR-Egger regression showed that the analysis was not affected by horizontal pleiotropy (P > 0.05) (**Table 2**). The MR-PRESSO analysis did not detect any significant pleiotropy or outliers (P > 0.05), as shown in **Table 3**. Additionally, funnel plots and “leave-one-out” analyses uncovered no significant outliers (**Figures 2C, D**, **3C, D**; **Supplementary Figures 3**, **4**), further substantiating the consistency and dependability of our findings.

**Table 2:** Heterogeneity and Pleiotropy between MMPs and DN.

Outcome	Exposure	Heterogeneity			Pleiotropy		
		Q statistic (IVW)	*P* value	FDR adjusted *P* value	MR-Egger Intercept	*P* value	FDR adjusted *P* value
Diabetic neuropathy	Matrix metalloproteinase-17 levels	5.85	0.75	0.75	0.0570	0.13	0.86
	Matrix metalloproteinase-9 levels	10.31	0.74	0.75	0.0112	0.61	0.86
	Matrix metalloproteinase-3 levels	9.16	0.10	0.50	0.0390	0.53	0.86
	Matrix metalloproteinase-2 levels	8.05	0.23	0.63	0.0375	0.52	0.86
	Matrix metalloproteinase-12 levels	0.83	0.36	0.72	NA	NA	NA
	Matrix metalloproteinase-14 levels	16.47	0.12	0.50	0.0167	0.74	0.86
	Matrix metalloproteinase-16 levels	7.76	0.73	0.75	0.0002	1.00	1.00
	Matrix metalloproteinase-7 levels	2.64	0.45	0.72	0.0480	0.52	0.86

NA, not applicable.

**Table 3:** Testing Pleiotropy of MMPs and DN using MRPRESSO.

Exposure	Outcome	Raw		Outlier corrected		Global P	Number of outliers	Distortion P
		OR (CI%)	*P*	OR (CI%)	*P*			
Matrix metalloproteinase-9 levels	Diabetic neuropathy	0.90 (0.82 - 0.99)	0.05	NA	NA	0.760	NA	NA
Matrix metalloproteinase-17 levels		1.00 (0.90 - 1.12)	0.95	NA	NA	0.729	NA	NA
Matrix metalloproteinase-2 levels		0.88 (0.79 - 0.99)	0.07	NA	NA	0.329	NA	NA
Matrix metalloproteinase-16 levels		1.15 (1.03 - 1.29)	0.03	NA	NA	0.743	NA	NA
Matrix metalloproteinase-14 levels		0.91 (0.75 - 1.10)	0.34	NA	NA	0.174	NA	NA
Matrix metalloproteinase-7 levels		1.02 (0.89 - 1.18)	0.77	NA	NA	0.520	NA	NA
Matrix metalloproteinase-3 levels		0.99 (0.87 - 1.13)	0.92	NA	NA	0.205	NA	NA

NA, not applicable.

## 4 Discussion

This study explored the causal relationship between MMPs and DN through two-sample MR analysis. IVW results indicated a negative genetic causal relationship between MMP-2 levels and the risk of DN, while MMP-16 levels showed a positive genetic correlation with the risk of DN. No significant causal associations were observed between other MMPs, including MMP-12, MMP-14, MMP-17, MMP-3, MMP-7, and MMP-9, and DN. This research contributes to the understanding of MMPs’ role in DN by employing a genetics-based approach, which is relatively unexplored in the existing literature. The findings from this research add a valuable genetics-based perspective to the understanding of MMPs’ role in DN, an area that has been relatively underexplored in existing literature. By identifying the differential impacts of specific MMPs on DN, this study underscores the clinical relevance of these molecular markers. The results could inform future therapeutic strategies, particularly by targeting MMP-2 and MMP-16, thus enhancing the potential for clinical translation of genetic findings into practice.

MMPs, a family of 23 endopeptidases, can degrade and remodel extracellular matrix proteins, participating in many physiological and pathological processes through regulating tissue remodeling, inflammatory responses, and cell migration (21). In the context of diabetes, high glucose treatment can damage the electron transport chain in mitochondria, leading to the production of reactive oxygen species (ROS), both high glucose and ROS can induce the expression of MMPs (22). In DN, changes in MMPs expression and activity can affect extracellular matrix remodeling, inflammatory responses, and the processes of nerve regeneration and damage, thereby participating in disease progression (23). Multiple substances, by inhibiting the activity of MMPs such as MMP-3 (24) and MMP-9 (25), exert antioxidant effects, which in turn inhibit neuroinflammation and neural damage. Thus, targeting specific MMPs is hypothesized to harness antioxidant actions to ameliorate DN. Increasing experimental evidence shows that MMPs such as MMP-2, MMP-9, and MMP-13 play roles in DN (26). For instance, in a streptozotocin-induced DN rat model, the activity of MMP-2 in the dorsal root ganglia and spinal cord decreased, and MMP-9 activity increased, significantly alleviating pathological pain by inhibiting MMP-9 or restoring MMP-2 activity (27). High glucose can upregulate ROS-mediated overexpression of MMP-13, inducing axonal degeneration, and inhibition of matrix metalloproteinase-13 can reverse neuropathy in diabetic mice (28). These studies suggest that MMPs are closely related to the disease progression of DN, but most are based on diabetic models, lacking in-depth research on the role of MMPs in the pathogenesis of DN.

To evaluate the consistency of our study results with clinical data, we analyzed results from previous clinical studies. A cross-sectional clinical study reported that the risk of DN is associated with elevated levels of serum MMP-2 (OR=4.5, p<10^-5) (29). Our findings diverge from this study, prompting further investigation into the reasons behind these differences. This deviation could be attributed to the smaller sample size and limited control over confounding factors in the previous study’s design. Specifically, it included 187 type 1 diabetes mellitus (T1DM) patients with diabetic peripheral neuropathy (DPN) as the experimental group—a relatively small cohort—and used T1DM patients without DPN as controls. In contrast, our study utilized a broader control group drawn from the general population. Furthermore, the DPN patients in the referenced study were, on average, 14 years older than the controls, introducing age as a potential confounding factor that could influence inflammatory markers such as MMPs. Conversely, an *in vivo* study suggested that MMP-2 overexpression might facilitate neurite growth, enhancing nerve regeneration in DN (30), thereby supporting the need for further investigation into MMP-2’s role in DN pathogenesis.

Regarding MMP-16, our results demonstrate a genetic correlation with DN risk. However, there is a notable gap in clinical studies directly examining the relationship between MMP-16 expression and DN. MMP-16, also known as MT3-MMP, is predominantly expressed in the nervous system, particularly in neural crest cell where it is implicated in the remodeling of the extracellular matrix (31). This enzyme is critical for cell migration in various tissues (32). Given the potential role of MMP-16 in the pathogenesis of neuropathy, it is imperative to further investigate its expression characteristics and mechanisms of action in clinical patients with DN. These insights could facilitate the development of MMP-16 as a biomarker for the severity of DN or as a therapeutic target.

Multiple clinical and basic experimental studies have indicated that MMP-9 is a risk factor for DN, and targeting inhibition of MMP-9 can alleviate DN-induced neuropathic pain and damage (33, 34). A study based on type 1 diabetic patients showed that peripheral blood MMP-9 levels might serve as surrogate biomarkers of retinopathy in type 1 diabetic patients free of other vascular complications (35). However, most studies between MMP-9 and DN have been based on rat models (33, 36, 37). In our study, IVW analysis results did not show statistical significance between MMP-9 and the risk of DN (P=0.061), but Multiplicative Random Effects analysis indicated a statistically significant causal relationship (OR=0.90, 95% CI: 0.82-0.99, P=0.029). The Multiplicative Random Effects model is often used when there is considerable heterogeneity among IVs, as it can account for the heterogeneity in instrument variable estimates. MR-PRESSO and Cochran’s Q tests indicated no significant overall heterogeneity, but it does not exclude that the results may reveal subtle genetic causal relationships not detected by the IVW method. Additionally, no genetic correlation was found between other MMPs and DN in this study, and these results await further verification by clinical data.

Despite this study using genetic variants as IVs through the MR method, applying extensive quality control and sensitivity analyses to reduce biases and insufficient control of confounding factors inherent in observational studies, thereby enhancing the reliability of the genetic causality. However, the study still has certain limitations: Firstly, not all MMPs were included in this analysis, which may limit the scope of our findings. Secondly, the study population primarily consisted of individuals of European descent, thus, the generalizability of the results to other ethnic groups remains to be established. Lastly, while this study provides insight into the genetic causality between MMPs and DN from a genetic perspective, the specific underlying mechanisms still require elucidation through basic research. Future research should include a wider range of MMPs, encompass diverse populations to validate the findings across different genetic backgrounds, and conduct mechanistic studies to directly link genetic associations with functional outcomes. Implementing longitudinal designs and clinical trials could further deepen our understanding of how MMPs influence DN progression and aid in developing targeted therapeutic strategies.

## 5 Conclusions

In conclusion, this study utilized the two-sample MR to investigate the genetic links between MMPs and DN, demonstrating the power of genetic tools in pinpointing risk factors for intricate diseases. We identified definitive genetic causal relationships for MMP-2 and MMP-16 with DN, shedding light on the molecular mechanisms that may underpin the pathogenesis of DN. These insights not only deepen our understanding of DN but also offer a foundation for crafting targeted therapeutic strategies that could mitigate or potentially reverse the progression of DN. Nonetheless, while these findings are encouraging, they highlight the need for further confirmation through studies involving larger and more ethnically diverse populations.

## Data Availability

The original contributions presented in the study are included in the article/**Supplementary Material**. Further inquiries can be directed to the corresponding author.

## Supplementary material

Supplementary Figure 1 forest plot of MMPs on DN: MMP-17 **(A)**, MMP-14 **(B)**, MMP-12 **(C)**, MMP-9 **(D)**, MMP-7 **(E)**, MMP-3 **(F)**.

Supplementary Figure 2 scatter plot of MMPs on DN: MMP-17 **(A)**, MMP-14 **(B)**, MMP-12 **(C)**, MMP-9 **(D)**, MMP-7 **(E)**, MMP-3 **(F)**.

Supplementary Figure 3 funnel plot of MMPs on DN: MMP-17 **(A)**, MMP-14 **(B)**, MMP-12 **(C**), MMP-9 **(D)**, MMP-7 **(E)**, MMP-3 **(F)**.

Supplementary Figure 4 leave-one-out analysis of MMPs on DN: MMP-17 **(A)**, MMP-14 **(B)**, MMP-12 **(C)**, MMP-9 **(D)**, MMP-7 **(E)**, MMP-3 **(F)**.
