# Supplementary figures and images for: Causal association between matrix metalloproteinases and diabetic neuropathy: a two-sample Mendelian randomization study
Source: Front Endocrinol (Lausanne). 2025 Jan 14;15:1429121. doi: 10.3389/fendo.2024.1429121 (PMC11772099; doi:10.3389/fendo.2024.1429121)

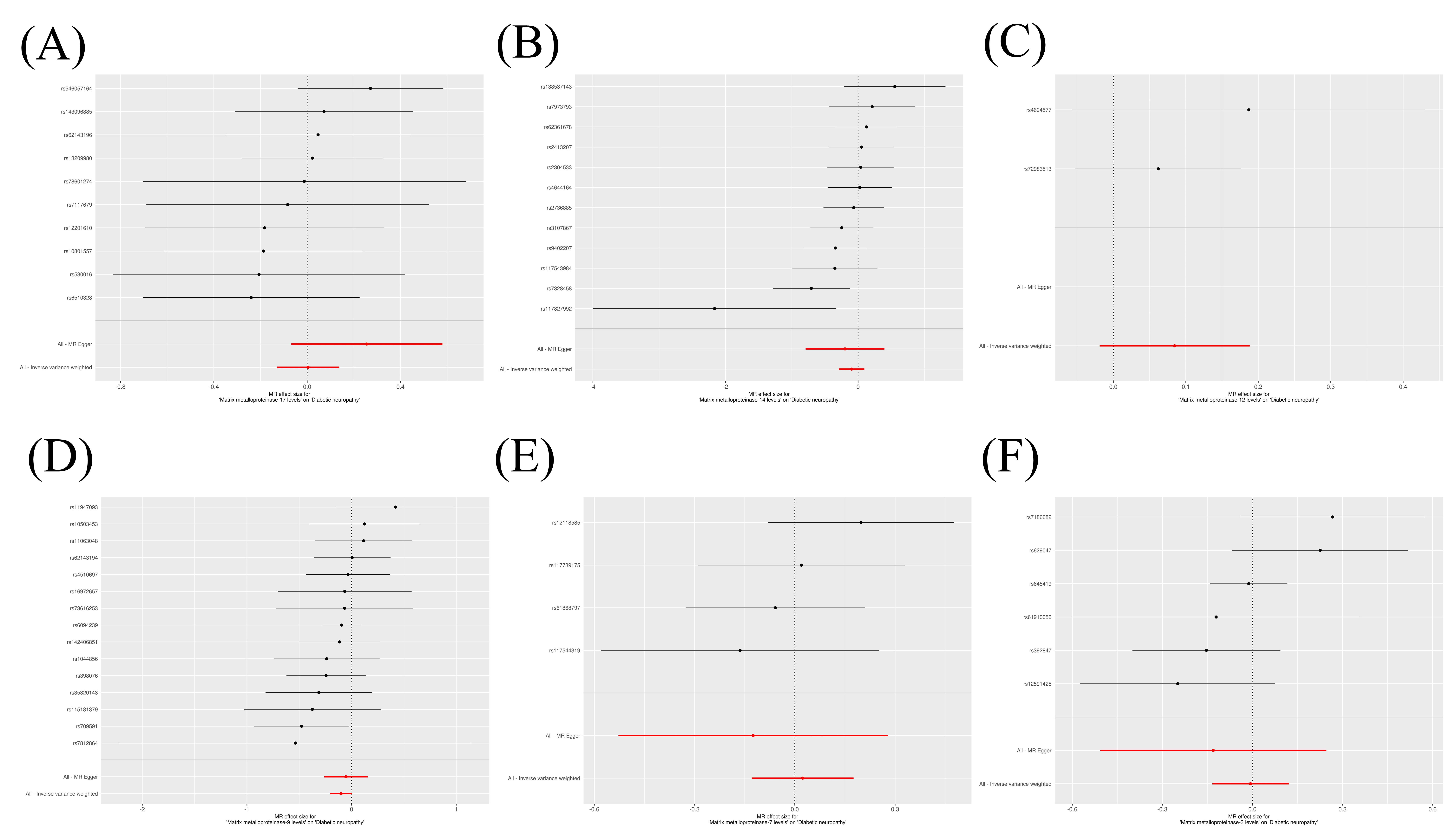

Supplement: Supplementary Figure 1 — forest plot of MMPs on DN: MMP-17 (A), MMP-14 (B), MMP-12 (C), MMP-9 (D), MMP-7 (E), MMP-3 (F). [file Image1.tif]

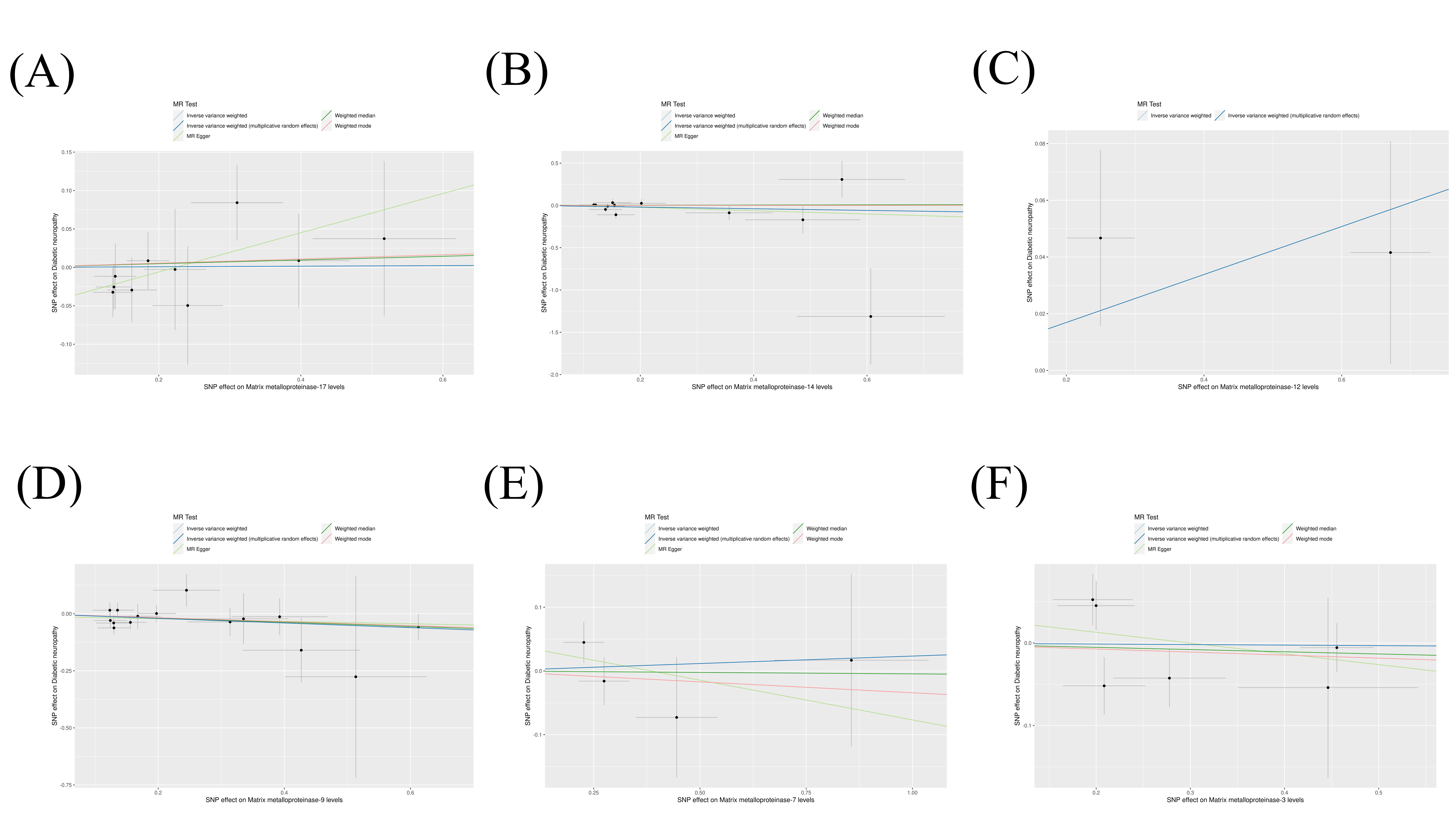

Supplement: Supplementary Figure 2 — scatter plot of MMPs on DN: MMP-17 (A), MMP-14 (B), MMP-12 (C), MMP-9 (D), MMP-7 (E), MMP-3 (F). [file Image2.tif]

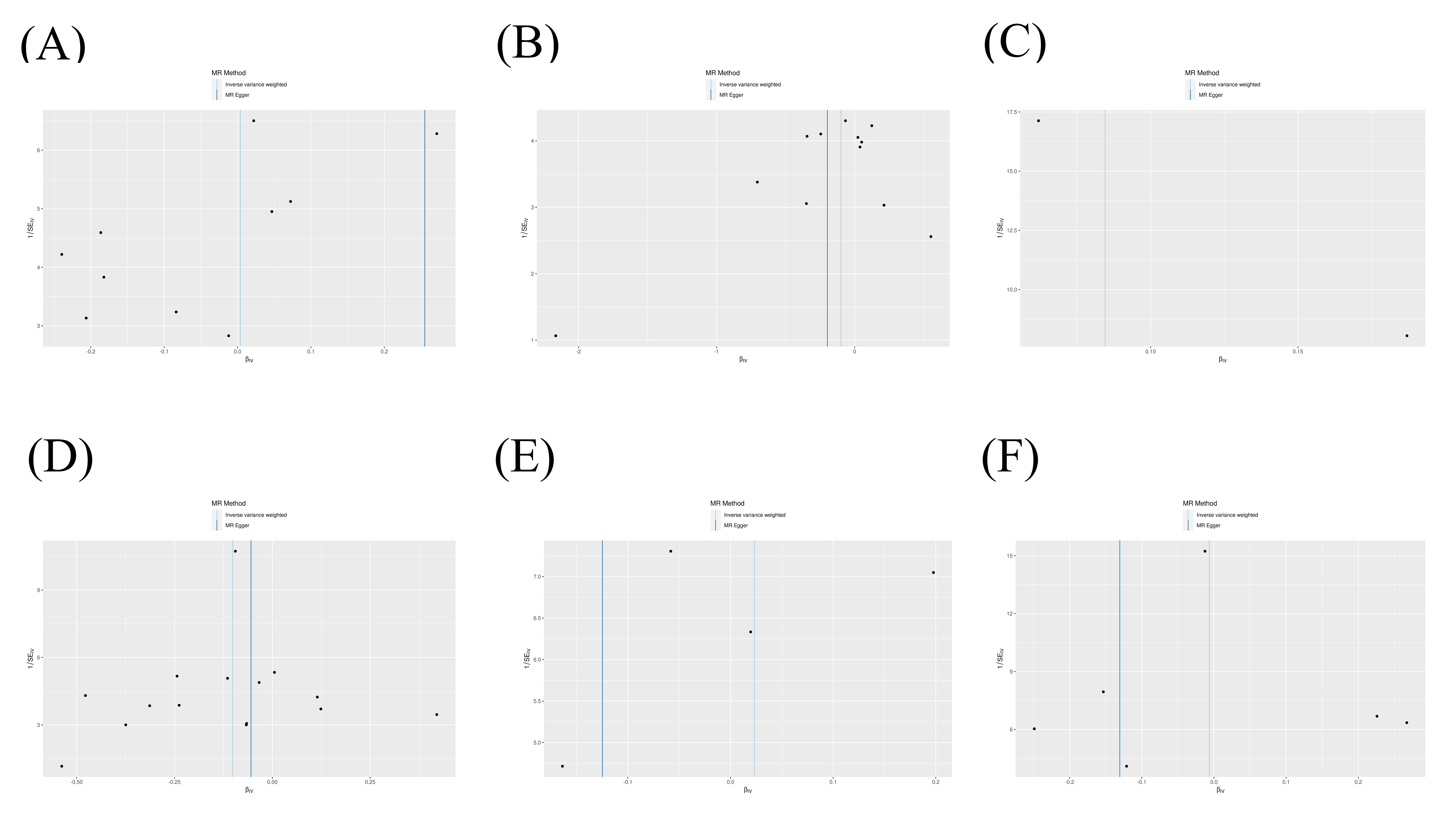

Supplement: Supplementary Figure 3 — funnel plot of MMPs on DN: MMP-17 (A), MMP-14 (B), MMP-12 (C), MMP-9 (D), MMP-7 (E), MMP-3 (F). [file Image3.tif]

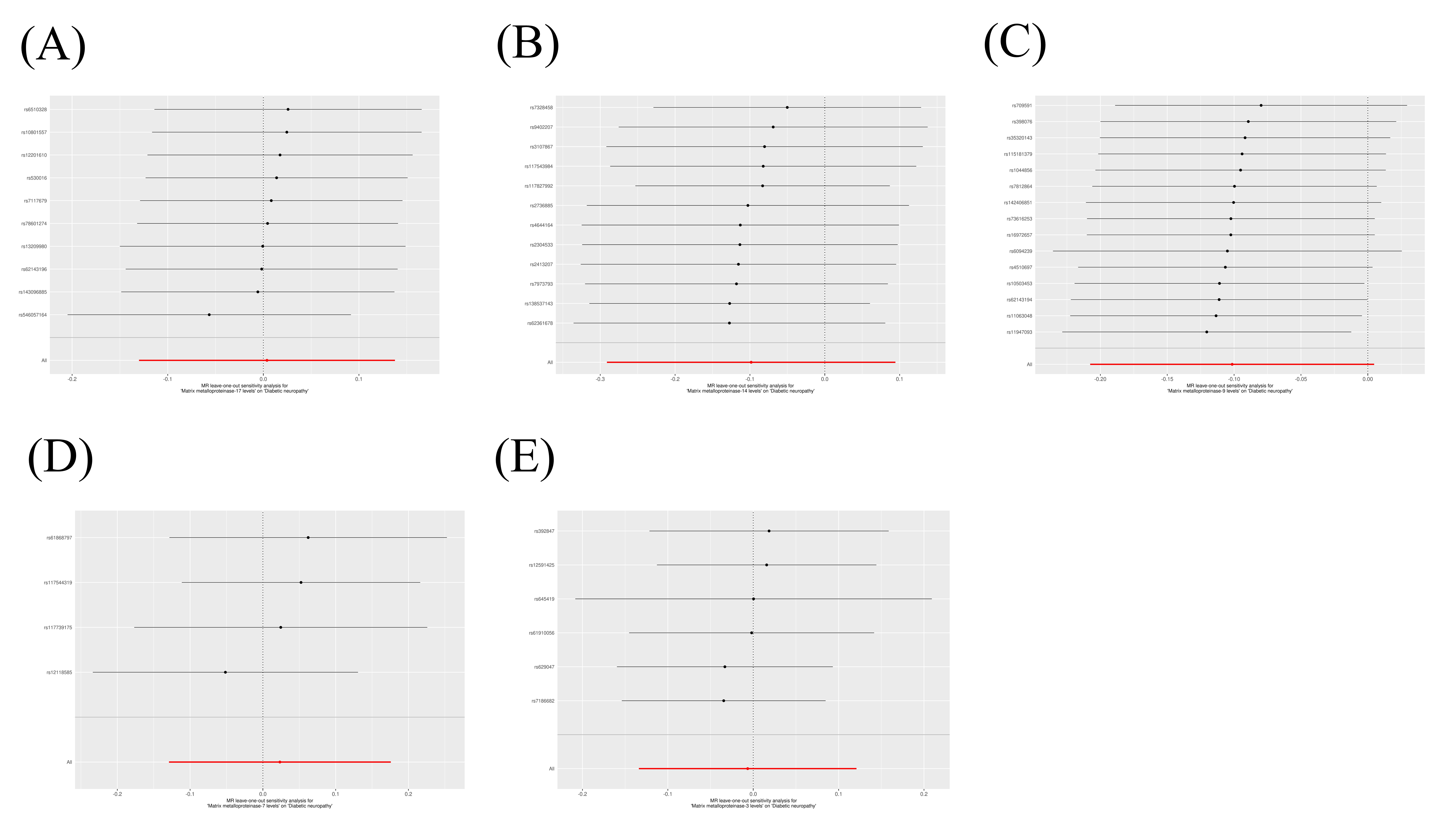

Supplement: Supplementary Figure 4 — leave-one-out analysis of MMPs on DN: MMP-17 (A), MMP-14 (B), MMP-12 (C), MMP-9 (D), MMP-7 (E), MMP-3 (F). [file Image4.tif]
