# Supplementary material for: Causal association between matrix metalloproteinases and diabetic neuropathy: a two-sample Mendelian randomization study
Source: Front Endocrinol (Lausanne). 2025 Jan 14;15:1429121. doi: 10.3389/fendo.2024.1429121 (PMC11772099; doi:10.3389/fendo.2024.1429121)
Supplement: Supplementary file 5 [file Table1.docx]

**Supplementary Table 1. Details of the GWASs included in the Mendelian randomization**

| **Character** | **Traits** | **GWAS ID** | **N（case/control）** | **Population** | **Number of SNPs** |
| --- | --- | --- | --- | --- | --- |
| Outcome | Diabetic neuropathy | DM_NEUROPATHY | 2,444/249,480 | European | NA |
| Exposure | Matrix metalloproteinase-14 levels (MMP14.5002.76.1) | GCST90241890 | 3,301 |  | NA |
| Exposure | Matrix metalloproteinase-16 levels (MMP16.5268.49.3) | GCST90241892 | 3,301 |  | NA |
| Exposure | Matrix metalloproteinase-17 levels (MMP17.2838.53.1) | GCST90241893 | 3,301 |  | NA |
| Exposure | Matrix metalloproteinase-9 levels (MMP9.2579.17.5) | GCST90241894 | 3,301 |  | NA |
| Exposure | Matrix metalloproteinase-12 levels | GCST90010152 | 1,301 |  | NA |
| Exposure | Matrix metalloproteinase-7 levels | GCST90010153 | 1,301 |  | 16,380,466 |
| Exposure | Matrix metalloproteinase-2 levels | GCST90010238 | 1,323 |  | NA |
| Exposure | Matrix metalloproteinase-3 levels | GCST90010239 | 1,323 |  | NA |
